# Lateral approach in laparoscopic cholecystectomy for acute cholecystitis with stones: advantages in reducing inflammation, stress, and enhancing recovery

**DOI:** 10.3389/fsurg.2026.1890877

**Published:** 2026-08-07

**Authors:** Yujie Zhao, Chunhong Li

**Affiliations:** Department of Gastroenterology, Songbei Campus, the Fourth Affiliated Hospital of Harbin Medical University, Harbin, China

**Keywords:** acute cholecystitis, gallstones, inflammatory markers, laparoscopic cholecystectomy, lateral approach, stress response, surgical trauma

## Abstract

**Background:**

Laparoscopic cholecystectomy is the preferred treatment for acute cholecystitis with gallstones; however, severe local inflammation, tissue adhesions, and unclear anatomical landmarks during the acute phase may increase surgical difficulty and operative trauma. Therefore, optimization of the surgical dissection approach remains clinically important.

**Objective:**

To explore the impact of different surgical approaches during laparoscopic cholecystectomy on perioperative recovery, inflammatory response, and stress state in patients with acute cholecystitis combined with gallstones, and to evaluate the clinical application value of the lateral approach vs. antegrade/retrograde dissection techniques.

**Methods:**

This retrospective controlled study reviewed the medical records of 150 patients with acute cholecystitis combined with gallstones who underwent laparoscopic cholecystectomy at our hospital from January 2023 to January 2025. Patients were not prospectively assigned to different surgical approaches; instead, eligible patients were retrospectively identified from existing medical records and grouped according to the surgical approach actually performed during laparoscopic cholecystectomy. The control group (*n* = 75) underwent antegrade/retrograde dissection laparoscopic cholecystectomy, while the observation group (*n* = 75) underwent lateral approach dissection laparoscopic cholecystectomy. The two groups were compared in terms of perioperative indicators (operative time, intraoperative blood loss, time to first flatus, time to first ambulation, drainage tube retention time, hospital stay), pain [visual analogue scale (VAS)] scores, levels of inflammatory markers [interleukin-6 (IL-6), interleukin-8 (IL-8), C-reactive protein (CRP), tumor necrosis factor-α (TNF-α)], stress response indicators [cortisol (Cor), adrenocorticotropic hormone (ACTH)], bile duct injury indicators [alkaline phosphatase (ALP), direct bilirubin (DBIL)], and incidence of complications.

**Results:**

The observation group had significantly lower intraoperative blood loss, drainage tube retention time, and hospital stay compared to the control group (*P* < 0.05). VAS score comparisons between the two groups showed significant differences in group effect (*F* = 10.413), time effect (*F* = 12.846), and interaction effect (*F* = 11.319) (*P* < 0.05); within-group analysis showed that VAS scores at 24 h and 48 h after surgery were significantly lower than those at 12 h after surgery in both groups, and VAS scores at 48 h were lower than those at 24 h (*P* < 0.05); between-group analysis showed that the observation group had lower VAS scores than the control group at 12 h, 24 h, and 48 h postoperatively (*P* < 0.05). At postoperative day 3, IL-6, IL-8, CRP, TNF-α, Cor, and ACTH levels were significantly higher than preoperative levels in both groups, while ALP and DBIL levels were lower than preoperative levels; the magnitude of change was more significant in the observation group (*P* < 0.05). There was no statistically significant difference in the incidence of complications between the two groups (10.7% vs. 8.0%, *P* > 0.05).

**Conclusion:**

Compared with antegrade/retrograde approaches, the lateral approach to laparoscopic cholecystectomy provides better minimally invasive advantages in treating acute cholecystitis with gallstones. It more effectively reduces intraoperative bleeding and postoperative drainage time, alleviates postoperative pain and physiological stress response, and facilitates rapid postoperative recovery, without increasing the risk of complications. It is worthy of further clinical promotion.

## Introduction

Acute cholecystitis is one of the most common acute abdominal conditions in clinical practice, with over 90% of cases accompanied by gallstones, presenting with pathological processes such as cystic duct obstruction, bacterial infection, and gallbladder wall edema (1, 2). Typical clinical manifestations include persistent severe pain in the right upper quadrant, fever, elevated white blood cell count, and abnormal liver function. If not promptly treated, the condition may progress to gangrenous cholecystitis, perforation, or even diffuse peritonitis, posing a serious threat to life (3). Therefore, early diagnosis and timely, effective treatment are essential for reducing mortality and improving prognosis. With the advancement of minimally invasive techniques, laparoscopic cholecystectomy has become the preferred treatment for acute cholecystitis and gallstones. Compared with traditional open surgery, LC offers advantages such as less trauma, faster postoperative recovery, shorter hospital stay, and better cosmetic outcomes, aligning with the modern surgical concept of “fast-track recovery” (4, 5). However, in the acute phase, significant pericholecystic inflammatory exudation and severe tissue adhesions increase the surgical difficulty and risk of complications (6). Thus, optimizing the intraoperative dissection pathway has become a key factor in improving surgical safety and efficacy.

Currently, common laparoscopic cholecystectomy approaches in clinical practice include antegrade dissection, retrograde dissection, and the lateral approach technique.

Although antegrade/retrograde dissection techniques are relatively well-established, they carry a higher risk of bile duct injury and intraoperative bleeding when inflammation is severe and anatomical landmarks are unclear (7). In contrast, the recently emerging lateral approach technique emphasizes entering the dissection plane from the lateral edge of the gallbladder body, thus avoiding the most inflamed Calot's triangle region. This facilitates better surgical visibility and safety and reduces intraoperative trauma (8). Relevant studies (9) have also suggested that this method may have potential advantages in reducing postoperative pain and shortening recovery time. However, its specific impact on inflammation, stress, and biliary system physiological indicators still lacks systematic evaluation. Based on this, the present study focuses on patients with acute cholecystitis and gallstones to compare the clinical efficacy of the lateral approach vs. antegrade/retrograde dissection in laparoscopic cholecystectomy, with particular attention to their effects on postoperative inflammatory marker levels, stress hormone changes, and bile duct injury-related indicators, aiming to provide a more scientific basis for the selection of surgical approaches and to promote the optimization and standardization of minimally invasive gallbladder surgery.

## Materials and methods

### Study design and patient source

This study was a retrospective controlled study. Patients were not prospectively assigned to different surgical approaches. Instead, the electronic medical records and operative records of patients with acute cholecystitis combined with gallstones who underwent laparoscopic cholecystectomy in the Department of General Surgery of our hospital from January 2023 to January 2025 were retrospectively reviewed. After applying the inclusion and exclusion criteria, 150 eligible patients with complete clinical data were included in the final analysis, including 75 patients who underwent conventional antegrade/retrograde dissection laparoscopic cholecystectomy and 75 patients who underwent lateral approach laparoscopic cholecystectomy. The sample size was based on the number of eligible patients with complete data available during the predefined study period, rather than on a formal *a priori* sample size or power calculation. Inclusion criteria: (1) All met the diagnostic criteria for acute cholecystitis with gallstones according to the Tokyo Guidelines 2018 (10): typical symptoms, positive Murphy's sign on physical examination, ultrasound indicating enlarged gallbladder, thickened gallbladder wall (>3 mm), presence of gallstones, and white blood cell count >10.0 × 10^9^/L; (2) Onset time within 72 h, and condition suitable for elective or emergency laparoscopic surgery; (3) Age 18–75 years, no restriction on sex; (4) No severe dysfunction of heart, lung, liver, or kidney before surgery, and able to tolerate anesthesia and surgical procedure; (5) Complete medical records and ability to cooperate with follow-up and related examinations. Exclusion criteria: (1) Intraoperative findings of gallbladder perforation, gangrene, or obvious common bile duct stones; (2) Combined with other gallbladder diseases such as biliary tumors or gallbladder polyps; (3) Severe coagulation dysfunction or immune system diseases; (4) Pregnant women or individuals with psychiatric disorders who could not cooperate. According to the different surgical approaches, patients were divided into a control group (*n* = 75) and an observation group (*n* = 75). The control group underwent conventional antegrade/retrograde dissection laparoscopic cholecystectomy, while the observation group underwent surgery using the lateral approach dissection technique. This study was approved by the Medical Ethics Committee of our hospital (Ethics No.: PWLC2417). All legal guardians of the patients were informed about the study content and signed informed consent forms. The study was conducted in accordance with the ethical principles of the Declaration of Helsinki.

### Surgical methods

All patients in this study completed routine preoperative assessment and preparation. General anesthesia was used during surgery, with the patient in a supine position, head elevated, feet lowered, and slightly tilted to the left to facilitate exposure of the right upper abdominal operative area. A 10 mm trocar was inserted through a vertical or transverse incision at the umbilicus to establish pneumoperitoneum, maintaining intra-abdominal pressure at 12–14 mmHg, and a laparoscope was inserted to examine the gallbladder and surrounding tissues.

The observation group underwent three-port laparoscopic cholecystectomy. In addition to the umbilical observation port, a 5 mm operating port was established approximately 2 cm below the xiphoid process, and another 5 mm auxiliary port was established approximately 2 cm below the costal margin at the right midclavicular line, forming a “Y-shaped” triangular operation area. Intraoperatively, by upward and rightward traction at a point near the base of the gallbladder body, the anatomical space between the gallbladder and liver was exposed, avoiding the severely inflamed Calot's triangle region. Dissection was performed in a lateral-to-medial direction layer by layer. The dissection began from the left side of the gallbladder body and gradually proceeded to the right edge of the gallbladder bed, following the subcapsular fibrous membrane layer of the liver for fine separation. An electrocautery hook was used for hemostasis and tissue separation during the procedure to reduce bleeding from the gallbladder bed and improve clarity of the surgical field. After clear identification of anatomical structures, the cystic duct and cystic artery were dissected and managed with titanium clips before being cut. The gallbladder was then completely removed. If the gallbladder was tense or contained numerous stones, decompression via puncture was performed to facilitate subsequent dissection.

For the control group, depending on the intraoperative anatomy and degree of inflammation, the surgeon selected antegrade or retrograde dissection techniques for conventional laparoscopic cholecystectomy. In the antegrade technique, dissection began from Calot's triangle, where the cystic duct and artery were first exposed and managed. The gallbladder was then dissected from the cystic duct end toward the fundus. In the retrograde technique, the gallbladder fundus was first retracted and exposed, and dissection proceeded from the fundus along the gallbladder bed toward the cystic duct, with the Calot's triangle dissected and associated structures managed last. Both techniques aimed to ensure clear exposure, accurate anatomical identification, and avoidance of bile duct injury.

Intraoperative bleeding was managed using electrocautery, spray coagulation devices, or gelatin sponge compression. In cases with significant bleeding from the gallbladder bed or risk of biliary injury, a drainage tube was placed via the auxiliary port under the right costal margin. After thorough irrigation of the abdominal cavity and confirmation of no active bleeding or bile leakage, the incisions were closed in layers.

Postoperatively, both groups received standardized perioperative management, including routine intravenous antibiotics (e.g., cephalosporins), proton pump inhibitors for acid suppression, fluid and electrolyte balance maintenance, and thrombosis prevention. On postoperative day 1, patients were encouraged to ambulate early. The nature and volume of drainage fluid were closely monitored, dietary structure was adjusted according to recovery status, and patients were gradually guided to resume eating and assessed for recovery. The decision to remove the drainage tube was made based on drainage volume and the patient's clinical presentation.

### Observation indicators and evaluation methods

1. **Perioperative indicators:** including operative time (from skin incision to closure), intraoperative blood loss (based on surgical records and drainage collection), time to first flatus, time to first ambulation, drainage tube retention time, length of hospital stay (days from surgery to discharge), and conversion to open surgery.
2. **Postoperative pain scores:** evaluated using the Visual Analogue Scale (VAS) (11) at 12 h, 24 h, and 48 h after surgery. The total score is 10, with higher scores indicating more severe pain.
3. **Inflammatory markers:** peripheral venous blood was collected before surgery and on postoperative day 3. Enzyme-linked immunosorbent assay (ELISA) was used to detect serum levels of interleukin-6 (IL-6), interleukin-8 (IL-8), C-reactive protein (CRP), and tumor necrosis factor-alpha (TNF-α).
4. **Stress response markers:** serum cortisol (Cor) and adrenocorticotropic hormone (ACTH) levels were measured before surgery and on postoperative day 3, using the same methods as above.
5. **Bile duct injury-related indicators:** serum alkaline phosphatase (ALP) and direct bilirubin (DBIL) levels were measured before surgery and on postoperative day 3 using an automated biochemical analyzer.
6. **Postoperative complications:** included bile leakage, incision infection, bile duct injury, intra-abdominal effusion, etc.

### Statistical methods

GraphPad Prism 8 was used for plotting, and SPSS 22.0 software was used for data analysis. The normality of continuous variables was assessed using the Shapiro–Wilk test combined with visual inspection of histograms and Q-Q plots. Continuous variables that approximately conformed to a normal distribution were expressed as mean ± standard deviation (x ± s). Comparisons between two independent groups were performed using independent-sample t-tests, and within-group comparisons before and after surgery were performed using paired t-tests. Repeatedly measured variables, such as VAS scores at different postoperative time points, were analyzed using repeated measures analysis of variance. Count data were expressed as percentages (%) and analyzed using the chi-square (*χ*²) test or Fisher's exact test, as appropriate. *P* < 0.05 was considered statistically significant.

## Results

### Comparison of baseline data

There were no statistically significant differences between the two groups in terms of gender, age, body mass index (BMI), time from onset to admission, type of gallstones, or presence of chronic comorbidities (*P* > 0.05), indicating comparability see Table 1.

**Table 1:** Comparison of baseline data.

Characteristic	Control (*n* = 75)	Observation (*n* = 75)	*t/x* ^2^	*P*
Gender	—	—	0.242	0.622
Male	32 (42.7%)	35 (46.7%)	—	—
Female	43 (57.3%)	40 (53.3%)	—	—
Age (years)	45.38 ± 8.56	44.97 ± 7.85	0.305	0.760
BMI (kg/m²)	24. 13 ± 2.26	23.85 ± 2.39	0.737	0.462
Time from onset to admission (h)	26.93 ± 4.28	27.09 ± 4.12	0.233	0.815
Type of stones	—	—	0.240	0.623
Single	41 (54.7%)	38 (50.7%)	—	—
Multiple	34 (45.3%)	37 (49.3%)	—	—
Comorbid chronic diseases	—	—	0.529	0.467
Yes	19 (25.3%)	23 (30.7%)	—	—
No	56 (74.7%)	52 (69.3%)	—	—

### Comparison of perioperative indicators

The intraoperative blood loss, duration of drainage, and length of hospital stay in the observation group were significantly less than those in the control group (*P* ＜ 0.05). There were no significant differences in operative time, time to first flatus, or time to ambulation between the two groups (*P* > 0.05). No patient in either group required conversion to open surgery during laparoscopic cholecystectomy (0/75 vs. 0/75) see Table 2.

**Table 2:** Comparison of perioperative indicators.

Outcome measure	Control (*n* = 75)	Observation (*n* = 75)	*t/x²*	*P*
Operative time (min)	67.41 ± 12.36	65.73 ± 11.95	0.846	0.398
Intraoperative blood loss (mL)	76.20 ± 15.84	62.84 ± 13.47	5.564	<0.001
Time to first flatus (h)	21.68 ± 4.75	20.93 ± 4.58	0.984	0.326
Time to ambulation (h)	44.45 ± 10.02	43.51 ± 9.78	0.581	0.561
Drainage duration (d)	3.85 ± 1.12	2.74 ± 0.95	6.545	<0.001
Hospital stay (d)	6.84 ± 1.57	5.31 ± 1.39	6.318	<0.001
Conversion to open surgery	0 (0.0%)	0 (0.0%)	0.000	1.000

### Comparison of pain levels

**Figure 1:**
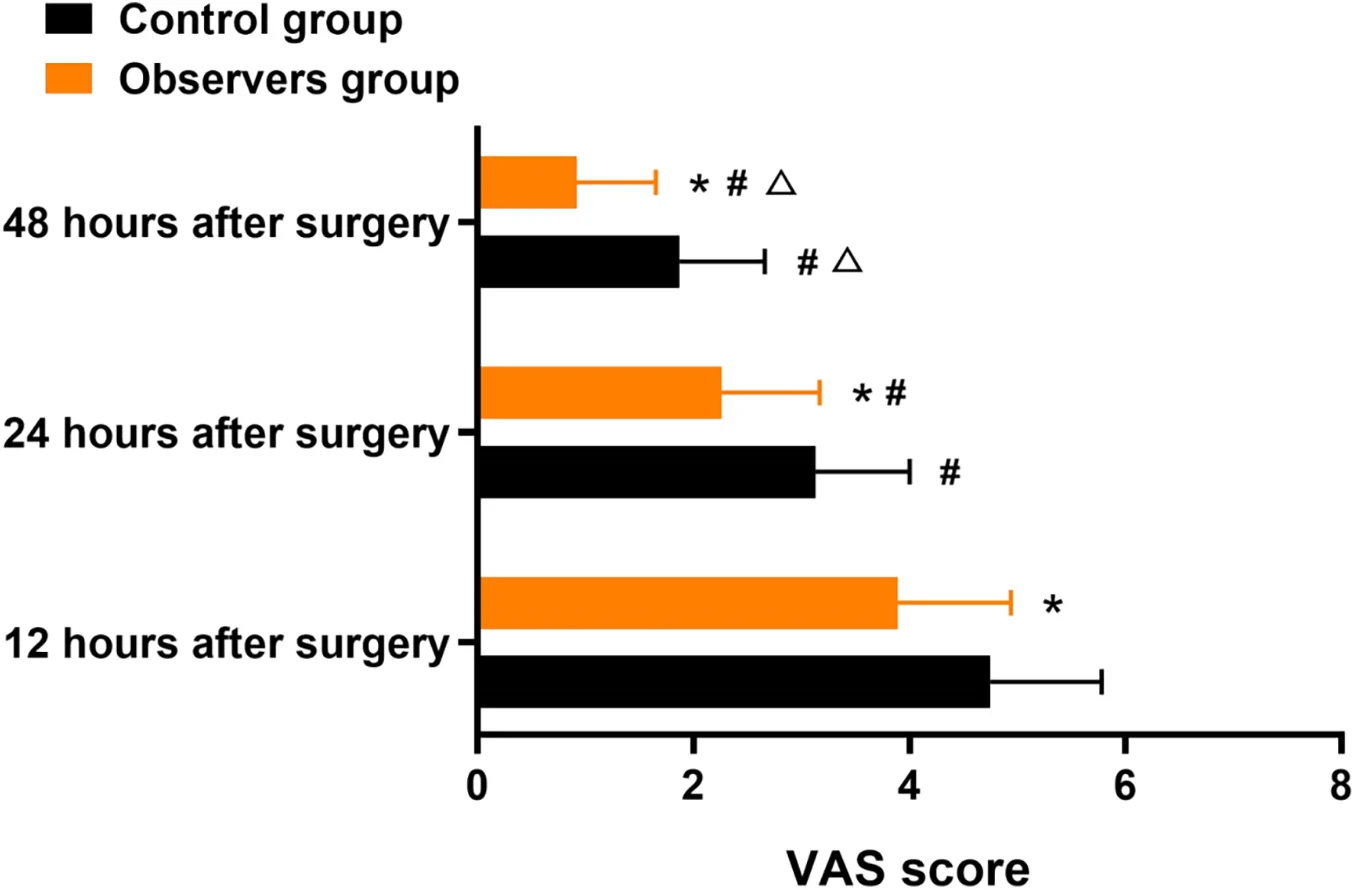
Comparison of pain levels. Compared with control group at the same time point, *P < 0.05; compared with 12 h post-op in same group, #*P* < 0.05; compared with 24 h post-op in same group, Δ*P* < 0.05.

Group effect (*F* = 10.413), time effect (*F* = 12.846), and interaction (*F* = 11.319) for VAS scores were statistically significant (*P* ＜ 0.05). Within-group comparisons: In both groups, VAS scores at 24 h and 48 h postoperatively were lower than those at 12 h, and scores at 48 h were lower than at 24 h (*P* ＜ 0.05). Between-group comparisons: At 12 h, 24 h, and 48 h postoperatively, VAS scores were significantly lower in the observation group than in the control group (*P* ＜ 0.05) see Figure 1.

### Comparison of inflammatory response indicators

At 3 days postoperatively, IL-6, IL-8, CRP, and TNF-α levels increased in both groups compared to preoperative levels, with significantly greater changes observed in the observation group (*P* ＜ 0.05) see Table 3.

**Table 3:** Comparison of inflammatory response indicators.

Indicator	Control (*n* = 75)	Observation (*n* = 75)	*t*	*P*
IL-6 (pg/mL)	—	—	—	—
Pre-op	25.73 ± 6.15	25.92 ± 5.93	0.192	0.847
Post-op 3 d	68. 15 ± 10.47^b^	54.32 ± 9.36^b^	8.528	<0.001
IL-8 (pg/mL)	—	—	—	—
Pre-op	42.57 ± 7.63	43.01 ± 7.54	0.355	0.722
Post-op 3 d	92.63 ± 13.76^b^	75.28 ± 12.04^b^	8.217	<0.001
CRP (mg/L)	—	—	—	—
Pre-op	12.24 ± 3.15	12.01 ± 3.42	0.428	0.669
Post-op 3 d	37.82 ± 6.97^b^	28.64 ± 5.48^b^	8.966	<0.001
TNF-α (pg/mL)	—	—	—	—
Pre-op	19.78 ± 3.83	20.05 ± 3.76	0.435	0.663
Post-op 3 d	46.92 ± 7.61^b^	37.26 ± 6.93^b^	8.128	<0.001

Compared with pre-op in the same group

b *P* ＜ 0.05.

### Comparison of stress response indicators

**Figure 2:**
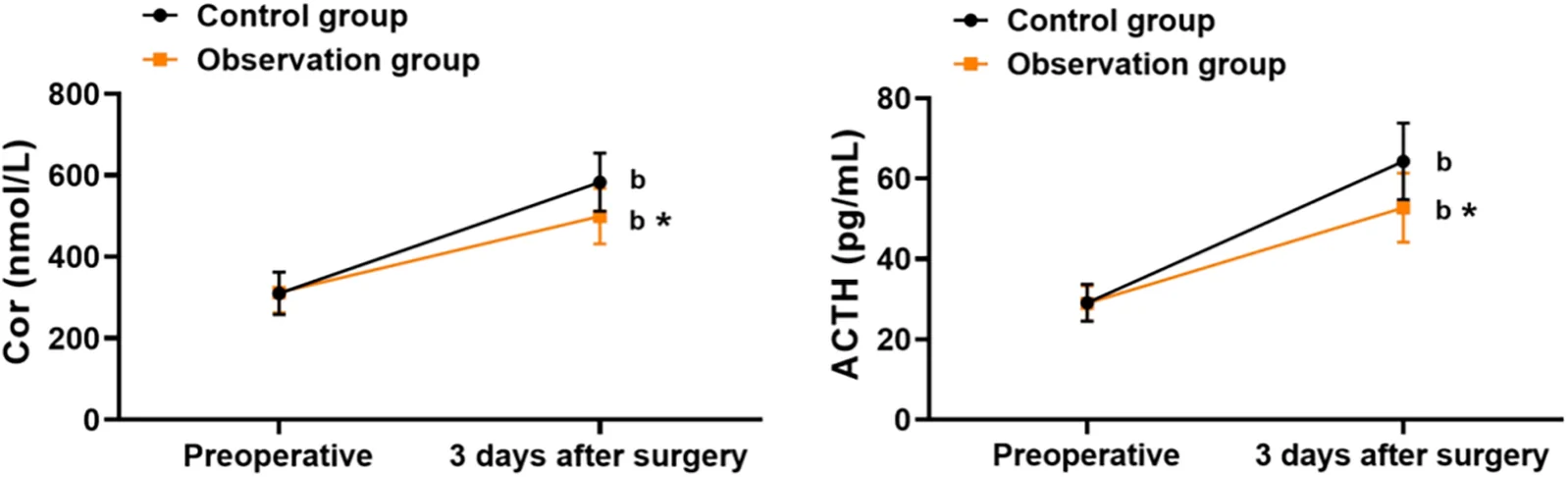
Comparison of stress response indicators. Compared with control group at same time point, *P ＜ 0.05; compared with same group pre-op, ^b^*P* ＜ 0.05.

Cor and ACTH levels at 3 days post-op were higher than pre-op in both groups, with greater increases in the observation group (*P* ＜ 0.05) see Figure 2.

### Comparison of bile duct injury indicators

**Figure 3:**
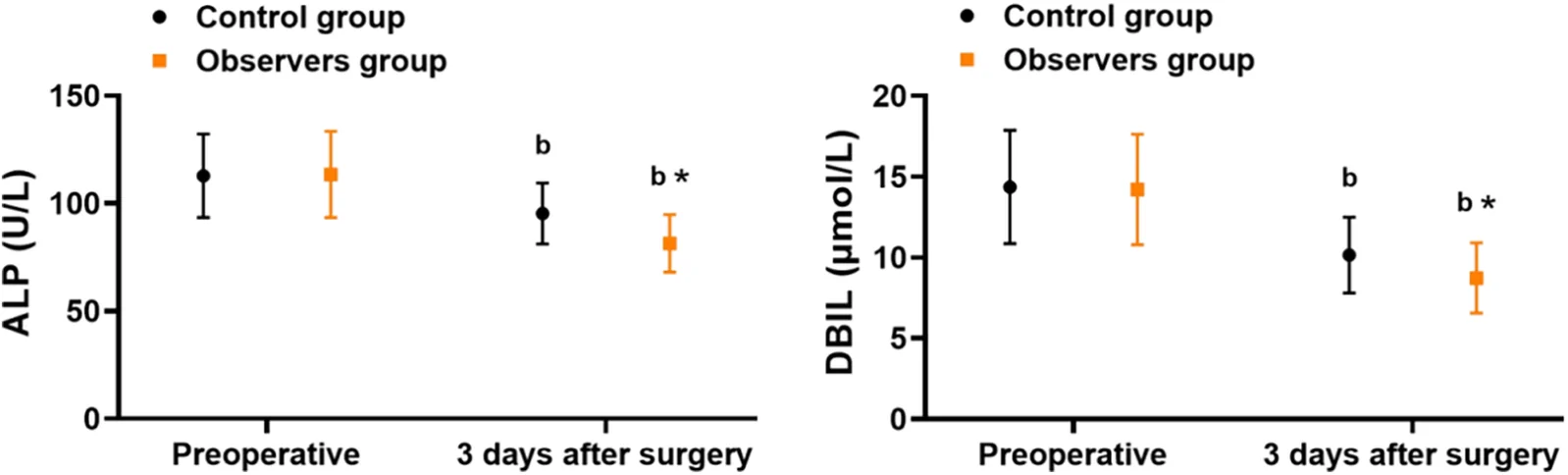
Comparison of bile duct injury indicators. Compared with control group at same time point, *P < 0.05; compared with same group pre-op, ^b^*P* < 0.05.

ALP and DBIL levels decreased at 3 days postoperatively in both groups, with more pronounced reductions in the observation group (*P* ＜ 0.05) see Figure 3.

### Comparison of postoperative complications

The incidence of postoperative complications was 8.0% (6/75) in the observation group and 10.7% (8/75) in the control group, with no statistically significant difference between the two groups (*P* ＞ 0.05) see Table 4.

**Table 4:** Comparison of postoperative complications.

Complication	Control (*n* = 75)	Observation (*n* = 75)	*X* ^2^	*P*
Bile leakage	2 (2.7%)	1 (1.3%)	—	—
Incisional infection	3 (4.0%)	2 (2.7%)	—	—
Bile duct injury	2 (2.7%)	2 (2.7%)	—	—
Abdominal effusion	1 (1.3%)	1 (1.3%)	—	—
Total incidence	8 (10.7%)	6 (8.0%)	0.315	0.574

## Discussion

The lateral approach, increasingly recognized in recent years as a viable laparoscopic cholecystectomy pathway, exhibits notable differences in intraoperative dissection strategy compared with traditional antegrade/retrograde methods. The conventional antegrade method typically involves gradual dissection from the gallbladder fundus toward the cystic duct and is better suited for cases with clearly defined surrounding anatomical structures and minimal adhesions (12). In contrast, the retrograde technique begins from the Calot's triangle, sequentially exposing the cystic duct and cystic artery, and is generally used in mild inflammation where local anatomy remains relatively intact (13). However, in cases of acute cholecystitis, the Calot's triangle is often the epicenter of severe inflammatory response, characterized by pronounced tissue edema, vascular engorgement, friable fatty tissue, and indistinct anatomical layers. These changes significantly increase the difficulty in identifying biliary structures and elevate the risk of bile duct injury and intraoperative bleeding (14, 15).

The lateral dissection technique circumvents the Calot's triangle by entering from the gallbladder's lateral margin or body, following a “lesion-avoidant, layer-by-layer” dissection strategy directed toward the cystic duct (16). This pathway helps the surgeon avoid severely inflamed regions, allowing for dissection within relatively clearer tissue planes. It also facilitates easier identification of the boundary between the gallbladder and hepatic bed and minimizes traction and damage to adjacent vessels and nerves (17). In this study, the intraoperative blood loss in the observation group was significantly lower than in the control group—an outcome attributable to the optimized dissection pathway. This suggests that the lateral approach not only allows more controlled operation under high-inflammation conditions but also potentially reduces damage to the local microvascular network, lowering the risk of intraoperative microbleeding. The lateral approach also demonstrated advantages in postoperative recovery. Data from this study showed that the observation group had superior outcomes regarding time to first bowel movement, time to first ambulation, duration of drainage tube placement, and total length of hospital stay compared with the control group. The underlying mechanisms may relate to reduced tissue traction and minimized local trauma during surgery. Histological studies (18) have shown that the extent of hepatic bed manipulation and damage to pericholecystic fatty tissue during cholecystectomy are closely linked to postoperative peritoneal exudation and inflammatory cytokine release. By minimizing direct electrocoagulation and traction on the liver bed, the lateral approach may reduce postoperative peritoneal inflammatory reactions and exudate formation, thus shortening drainage duration and hospitalization. Regarding pain management, the lateral approach also showed favorable results. In this study, the VAS scores at 12 h, 24 h, and 48 h postoperatively were significantly lower in the observation group than in the control group. Sources of postoperative pain include not only the incision but also electrocoagulation of the gallbladder bed, local tissue tension, and intraoperative traction (19). Reduced traction and thermal damage to the gallbladder bed during dissection may be key contributors to decreased postoperative pain in the lateral approach.

Surgical intervention, particularly involving visceral organs, often triggers complex stress response pathways (20). These responses include both local inflammatory activation and systemic neuroendocrine feedback, with the primary goal of maintaining compensatory homeostasis in the face of surgical trauma (21, 22).

Patients with acute cholecystitis are already in a pro-inflammatory state, and additional intraoperative trauma can amplify immune overreaction, potentially leading to systemic inflammatory response syndrome (SIRS) or impaired postoperative recovery (23). In terms of inflammation, surgical tissue damage, changes in vascular permeability, and neutrophil activation lead to a sharp increase in pro-inflammatory cytokine release (24). These mediators not only induce local immune cell aggregation and phagocytosis but also circulate to the hypothalamus, further stimulating the HPA axis and initiating a classic neuroendocrine-immune stress cascade (25). Results from this study showed that levels of inflammatory cytokines and stress hormones on postoperative day 3 were significantly higher than preoperative levels in both groups.

This confirms that even laparoscopic surgery, considered minimally invasive, inevitably provokes a certain degree of systemic physiological stress. This finding aligns with previous studies (26, 27) and suggests that surgery-induced stress is a ubiquitous phenomenon. Further comparison revealed that the increase in inflammatory markers and stress hormones was significantly lower in the lateral approach group, indicating a milder systemic physiological reaction to surgical trauma. Mechanistically, this may be attributed to the lateral approach's effective avoidance of the highly inflamed Calot's triangle during dissection, resulting in less disruption to local blood and lymphatic tissues. This limits the release of pro-inflammatory mediators and reduces HPA axis stimulation. This immunoprotective effect, grounded in reduced local trauma, aligns well with the “minimal immunological disturbance” concept increasingly emphasized in the emerging field of surgical immunology (28). Supporting literature concurs with these findings. For instance, Luo et al. reported in their comparative study of left hemicolectomy approaches (29) that the lateral approach, due to its lower risk of intraoperative complications, resulted in significantly lower postoperative IL-6 and TNF-α levels than the traditional pathway. This indicates a milder postoperative inflammatory response. Moreover, faster normalization of Cor and ACTH levels suggests a lighter burden on the HPA axis and promotes earlier reestablishment of endocrine homeostasis postoperatively. These differences are particularly important for high-risk populations (e.g., elderly patients, those with metabolic syndrome, or chronic inflammatory diseases), as their baseline stress tolerance is reduced, and excessive postoperative inflammatory or hormonal responses may trigger complications such as postoperative delirium, metabolic disorders, or cardiocerebrovascular events. Thus, selecting a surgical approach with lower stress burden may be of considerable importance in reducing postoperative complications and accelerating overall recovery. Additionally, it is worth noting that the observation group exhibited more pronounced postoperative reductions in ALP and DBIL, suggesting smoother postoperative bile drainage. This may be due to reduced traction and irritation of the cystic duct and common hepatic duct via the lateral pathway, thereby lowering the risk of transient postoperative biliary dysfunction. Although no statistical difference in bile duct injury rates was observed between the groups, from the perspective of “functional recovery,” the lateral approach may offer superior protection for the biliary system. This view is supported by findings from Song et al., who reported in their comparative study of surgical approaches for acute cholecystitis (30) that the lateral pathway reduced the incidence of postoperative jaundice and reflux cholangitis caused by biliary hypertension.

### Limitations and future perspectives

Although this study has achieved preliminary results in exploring the effects of different surgical approaches on postoperative inflammation and stress responses in patients with acute cholecystitis complicated by gallbladder stones, certain limitations exist and must be recognized. First, this was a single-center, retrospective study, and the influence of selection bias and information bias could not be completely avoided. Surgeons' choices regarding surgical pathways may have been influenced by subjective experience or differences in patient condition, thereby affecting the objectivity of the comparison. In addition, patients with gallbladder perforation or gangrenous cholecystitis were excluded from this study. Although this exclusion criterion was applied equally to both groups and therefore did not substantially affect the internal comparison between the two surgical approaches, it may limit the generalizability of the findings to patients with more severe or complicated acute cholecystitis. Whether the lateral approach can provide similar advantages in this subgroup requires further investigation.

Second, because this was a retrospective exploratory study, no formal *a priori* sample size or power calculation was performed. The sample size was based on the number of eligible patients with complete clinical data during the predefined study period. Therefore, future prospective studies with formal sample size estimation are needed to further confirm the robustness of the present findings. Third, limited by sample size and follow-up duration, this study focused primarily on short-term physiological changes after surgery and failed to systematically observe the full dynamic process of postoperative inflammation and stress responses across multiple postoperative time points. Additionally, long-term postoperative follow-up was not performed, and therefore the sustained impact of different surgical techniques on biliary function recovery could not be assessed. Although ALP and DBIL levels on postoperative day 3 showed a more pronounced decline in the lateral approach group, suggesting smoother bile drainage, this inference remains based on short-term observation and is insufficient to determine the long-term benefits of the surgical technique on biliary function. Furthermore, this study did not incorporate patient-reported recovery experiences or quality-of-life indicators, making it difficult to fully evaluate the overall benefit of the surgical pathway from the patient's perspective.

In future research, large-sample, multicenter, prospective studies should be conducted to further validate the safety and efficacy of the lateral approach. Future studies should also include longer follow-up of biliary function, dynamic monitoring of inflammatory and stress responses, and patient-reported outcomes. Moreover, subgroup analyses of patients with more severe inflammatory conditions, such as gallbladder perforation or gangrenous cholecystitis, may help clarify whether the lateral approach remains feasible and beneficial in more complex cases. These efforts may contribute to a more comprehensive evaluation system and promote the optimization and standardized clinical application of this surgical technique.

## Conclusion

The results of this study indicate that in patients with acute cholecystitis complicated by gallbladder stones, laparoscopic cholecystectomy performed via the lateral approach offers more pronounced minimally invasive advantages compared with traditional antegrade/retrograde dissection techniques. This surgical approach effectively reduces intraoperative blood loss, shortens postoperative drainage duration and hospital stay, alleviates postoperative pain, lowers levels of inflammatory markers and stress hormones, and promotes faster postoperative recovery without increasing the risk of bile duct injury or postoperative complications. Therefore, the lateral approach may have clinical value in enhancing the safety of laparoscopic cholecystectomy and improving postoperative recovery efficiency in selected patients with acute cholecystitis and gallstones.

## Data Availability

The original contributions presented in the study are included in the article/Supplementary Material, further inquiries can be directed to the corresponding author/s.
